# Novel *GNAI3* mutation in a Chinese family with auriculocondylar syndrome and treatment of severe dentofacial deformities: a 5-year follow-up case report

**DOI:** 10.1186/s12903-024-04575-1

**Published:** 2024-07-16

**Authors:** Yulin Shi, Liang Rong, Siying Liu, Yiwen Liu, Chunlin Zong, Jinbiao Lu, Hongtao Shang, Yang Xue, Lei Tian

**Affiliations:** 1 State Key Laboratory of Oral & Maxillofacial Reconstruction and Regeneration, National Clinical Research Center for Oral Diseases, Shaanxi Clinical Research Center for Oral Diseases, Department of Oral and Maxillofacial Surgery, School of Stomatology, The Fourth Military Medical University, 145 West Changle Road, Xi’an, 710032 PR China; 2 Department of Stomatology, Air Force Medical Center, The Fourth Military Medical University, 30 Fucheng Road, Beijing, 100089 China; 3 State Key Laboratory of Oral & Maxillofacial Reconstruction and Regeneration, National Clinical Research Center for Oral Diseases, Shaanxi Clinical Research Center for Oral Diseases, Department of Orthodontics, School of Stomatology, The Fourth Military Medical University, 145 West Changle Road, Xi’an, 710032 PR China; 4 State Key Laboratory of Oral & Maxillofacial Reconstruction and Regeneration, National Clinical Research Center for Oral Diseases, Shaanxi Clinical Research Center for Oral Diseases, Department of Oral Surgery, School of Stomatology, The Fourth Military Medical University, 145 West Changle Road, Xi’an, 710032 PR China

**Keywords:** Auriculocondylar syndrome, *GNAI3*, Dentofacial deformity, Distraction osteogenesis, Orthognathic surgery, 3D digital technology

## Abstract

**Background:**

Auriculocondylar syndrome (ARCND) is an extremely rare autosomal dominant or recessive condition that typically manifests as question mark ears (QMEs), mandibular condyle hypoplasia, and micrognathia. Severe dental and maxillofacial malformations present considerable challenges in patients’ lives and clinical treatment. Currently, only a few ARCND cases have been reported worldwide, but most of them are related to genetic mutations, clinical symptoms, and ear correction; there are few reports concerning the treatment of dentofacial deformities.

**Case presentation:**

Here, we report a rare case of ARCND in a Chinese family. A novel insertional mutation in the guanine nucleotide-binding protein alpha-inhibiting activity polypeptide 3 (*GNAI3)* was identified in the patient and their brother using whole-exome sequencing. After a multidisciplinary consultation and examination, sequential orthodontic treatment and craniofacial surgery, including distraction osteogenesis and orthognathic surgery, were performed using three-dimensional (3D) digital technology to treat the patient’s dentofacial deformity. A good prognosis was achieved at the 5-year follow-up, and the patient returned to normal life.

**Conclusions:**

ARCND is a monogenic and rare condition that can be diagnosed based on its clinical triad of core features. Molecular diagnosis plays a crucial role in the diagnosis of patients with inconspicuous clinical features. We present a novel insertion variation in *GNAI3,* which was identified in exon 2 of chromosome 110116384 in a Chinese family. Sequential therapy with preoperative orthodontic treatment combined with distraction osteogenesis and orthognathic surgery guided by 3D digital technology may be a practical and effective method for treating ARCND.

## Background

Auriculocondylar syndrome (ARCND; OMIM #602,483, #614,669, and #615,706) is an extremely rare autosomal dominant or recessive condition with a prevalence of less than 1 in 1,000,000 [1]. The typical triad manifestations of ARCND include question mark ears (QMEs), mandibular condyle hypoplasia, and micrognathia [2]. Other facial abnormalities [3] in the hearing [4], respiratory [5], digestive [6], reproductive [7], and even developmental [5] systems have also been reported. However, differences in gene expression have led to a wide range of differences in the clinical phenotypes between individuals and their families [6]. In addition, ARCND can be misdiagnosed as other diseases, including craniomaxillofacial deformities such as the oculo-auriculo-vertebral spectrum [7], Treacher Collins syndrome [8], mandibulofacial dysostosis [8], Guion-Almeida type, and Meier-Gorlin syndrome [9]. Ultimately, the varied individual clinical characteristics and extremely low incidence of ARCND contribute to the limited clinical knowledge of this condition.

Currently, fewer than 100 cases of ARCND have been reported in the literature [10]. However, most of them are related to genetic mutations, clinical symptoms, and ear correction in this disease, whereas correction of dentofacial deformities has rarely been reported [5, 11]. Therefore, clinicians must summarize individual clinical manifestations and report on feasible and effective clinical treatments. In this paper, we present the case of a Chinese patient with ARCND and the clinical features of her family member. A novel insertional mutation in the guanine nucleotide-binding protein alpha-inhibiting activity polypeptide 3 (*GNAI3)* was identified using whole-exome sequencing. Additionally, we performed sequential orthodontic treatment and craniofacial surgery, including distraction osteogenesis and orthognathic surgery, with the aid of digital technology, to treat the patient. A good prognosis was achieved, and the patient was satisfied with the treatment. Through this case report, we hope to improve our understanding of the genetic pathogenesis of ARCND and provide an effective clinical treatment for dentofacial malformations.

## Case presentation

**Fig. 1:**
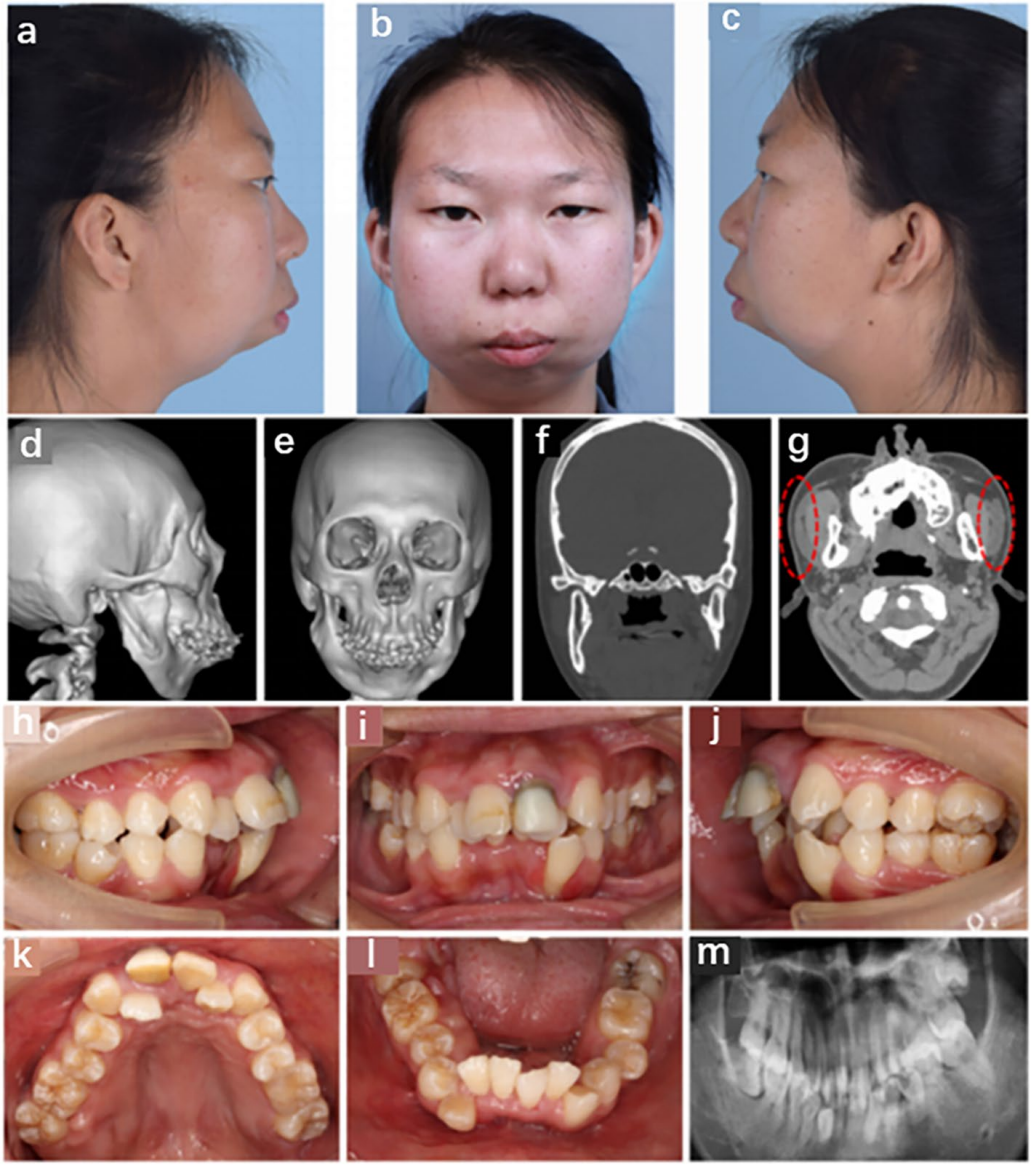
Phenotypic presentation of the patient. **a**-**c** The side view of the face showing micrognathia, full cheeks, and round face shape. **d**-**f** Three-dimensional (3D) reconstruction of computed tomography (CT) data indicating micrognathia, bony prominence of the mandibular rami, and mandibular condyle hypoplasia. **g** The red dashed lines indicate a heterotopic parotid gland. **h**–**l** Patient’s intraoral view showing deep the overbite and overjet of anterior teeth, disordered tooth, and crowded dental arch. **m** The panoramic radiograph shows severe malocclusion

A 23-year-old woman was admitted to our hospital with severe mandibular malformations and poor occlusion, accompanied by severe sleep snoring for more than 20 years. With increasing age, the deformity became increasingly severe. Physical examination of the patient revealed facial asymmetry, a round face with prominent cheeks (more pronounced on the right), retrognathia, and mandibular hypoplasia, presenting as a typical "beak deformity" (Fig. 1a-c). Computed tomography (CT) of the skull revealed marked hypoplasia of the mandibular body, rami, and both condyles (Fig. 1d-f). The bilateral parotid glands were small and ectopic towards the front edge of the masseter muscle (red dashed lines in Fig. 1g).

Intraoral examination revealed crowded teeth, malocclusion, and malalignment. The patient presented with an approximate Class I molar relationship, overbite, and overjet in the anterior teeth and a left-sided posterior crossbite (Fig. 1h-m). The interincisal distance was 30 mm, indicating limited mouth opening, but there was no clicking sound or tenderness in the area of bilateral temporomandibular joints (TMJs). No distinct cleft or notching between the lobe and helix was observed, and the morphology and function of the middle and inner ears were normal.

**Fig. 2:**
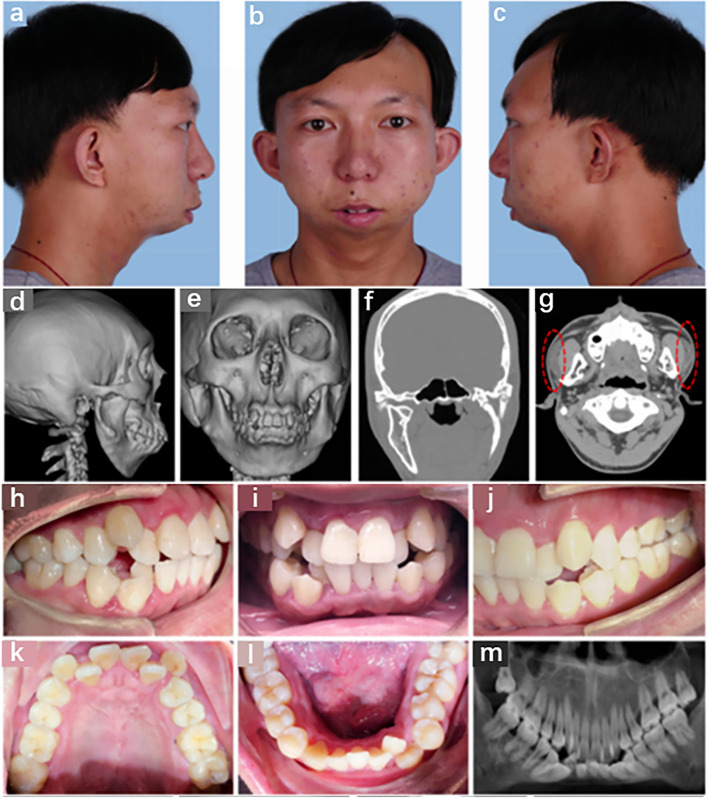
Phenotypic presentation of the patient’s younger brother. **a**-**c** The side view of the face showing similar micrognathia, full cheeks, and a round face shape. **d**-**f** Three-dimensional (3D) reconstruction of computed tomography (CT) data indicating micrognathia, bony ridge protruding on the mandibular rami, and mandibular condyle hypoplasia. **g** The red dashed lines indicate a heterotopic parotid gland. **h**–**l** Intraoral photographs showing the deep overbite and overjet of anterior teeth, disordered tooth and crowded dental arch. **m** The panoramic radiograph shows severe malocclusion

**Fig. 3:**
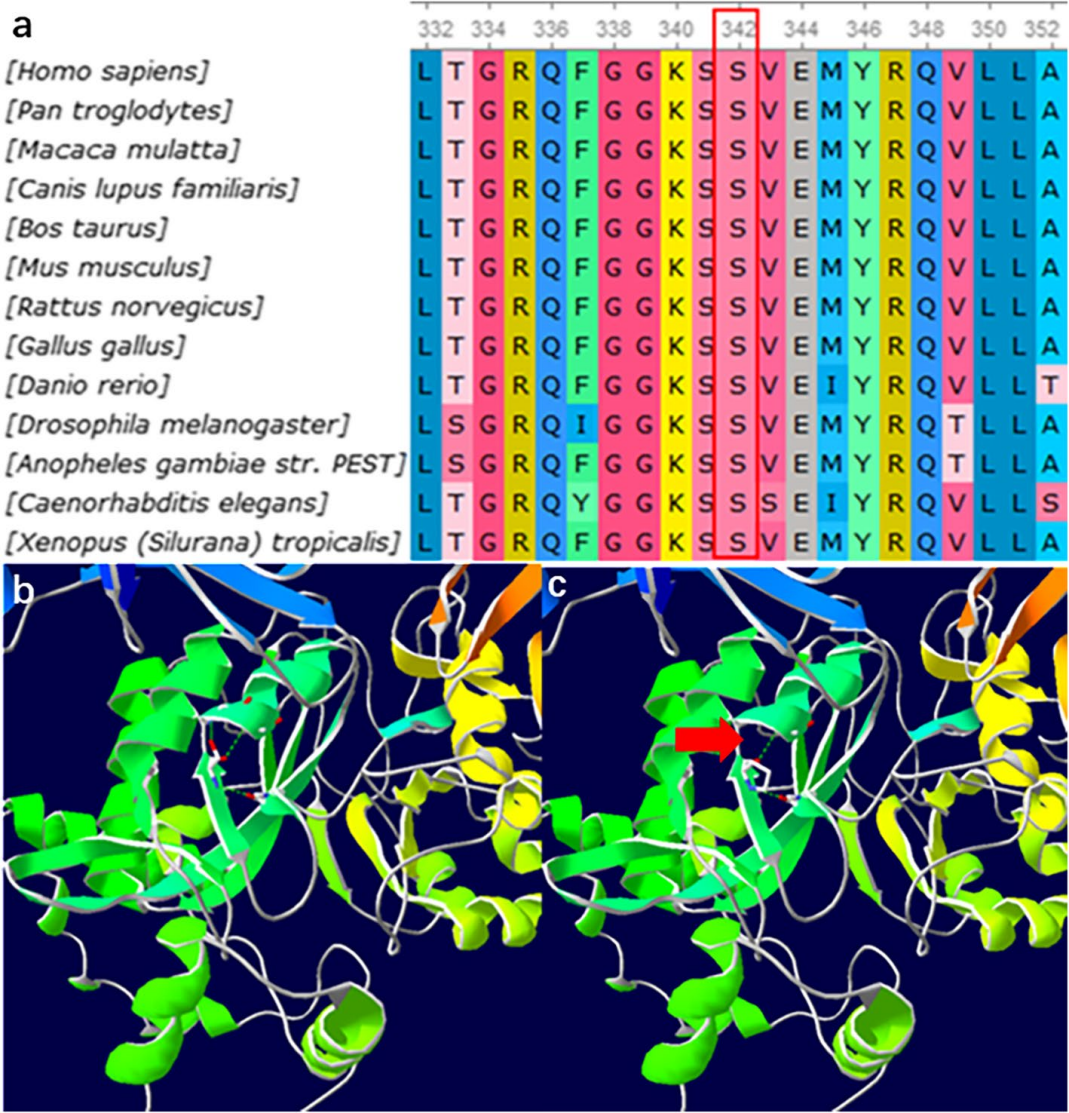
The *GNAI3* mutation contributed to the disruption of the hydrogen bond between serine 342 and glutamic acid 344. **a** Evaluation of amino acid conservation using Ugene. The serine indicated in the red box is highly conserved across various species. **b**, **c** Visualizing the three-dimensional (3D) molecular structure of protein–ligand using SWISS-MODEL. An overview of the wild-type (**b**) and mutant type (**c**), showing the disrupted hydrogen bond between serine 342 and glutamic Acid 344 (the red arrow)

Polysomnography revealed an apnea–hypopnea index of 10.8, indicating low-grade obstructive sleep apnea syndrome (OSAS) and moderate hypoxemia. The patient’s physical and mental development was normal, and she had no speech or learning problems. Assessment of the family history revealed that her younger brother had similar symptoms (Fig. 2), whereas the proband was healthy and did not exhibit any facial abnormalities. Whole-exome sequencing revealed that the patient and their younger brother harbored a novel insertional mutation in *GNAI3* (Table 1). Three-dimensional (3D) molecular structure prediction of the protein and disruption of hydrogen bonds are shown in Fig. 3.

**Table 1:** The results of whole-exome sequencing

Mutated gene	Chromosomal location	Transcript	Exon	Nucleotide change	Amino acid change	Diagnosis	Source of mutation
*GNAI3*	chr1:110,116,384	NM_006496	exon 2	c.144_145insCATTGTGAAACAGATGAA	p.T48_I49insHCETDE	ARCND I	Father

Based on the patient’s clinical presentation and molecular diagnosis, the patient was diagnosed with ARCND I, OSAS, and moderate hypoxemia. The principal problems to be addressed were convexity of the midface and micrognathia deformity, hypoplasia of the condylar region, retrognathism, and clockwise rotation of the mandible.In April 2016, after consulting with an orthodontist, a sequential treatment plan consisting of orthodontic treatment and craniofacial surgery with the aid of digital technology was established. During the preoperative orthodontic treatment, teeth 12 (located outside the lingual dental arch), 21 (with dead pulp), 33 (affected by severe periodontal disease), and 44 were extracted. Additionally, we aligned the crowded teeth and leveled the dentition to minimize tooth compensation.

**Fig. 4:**
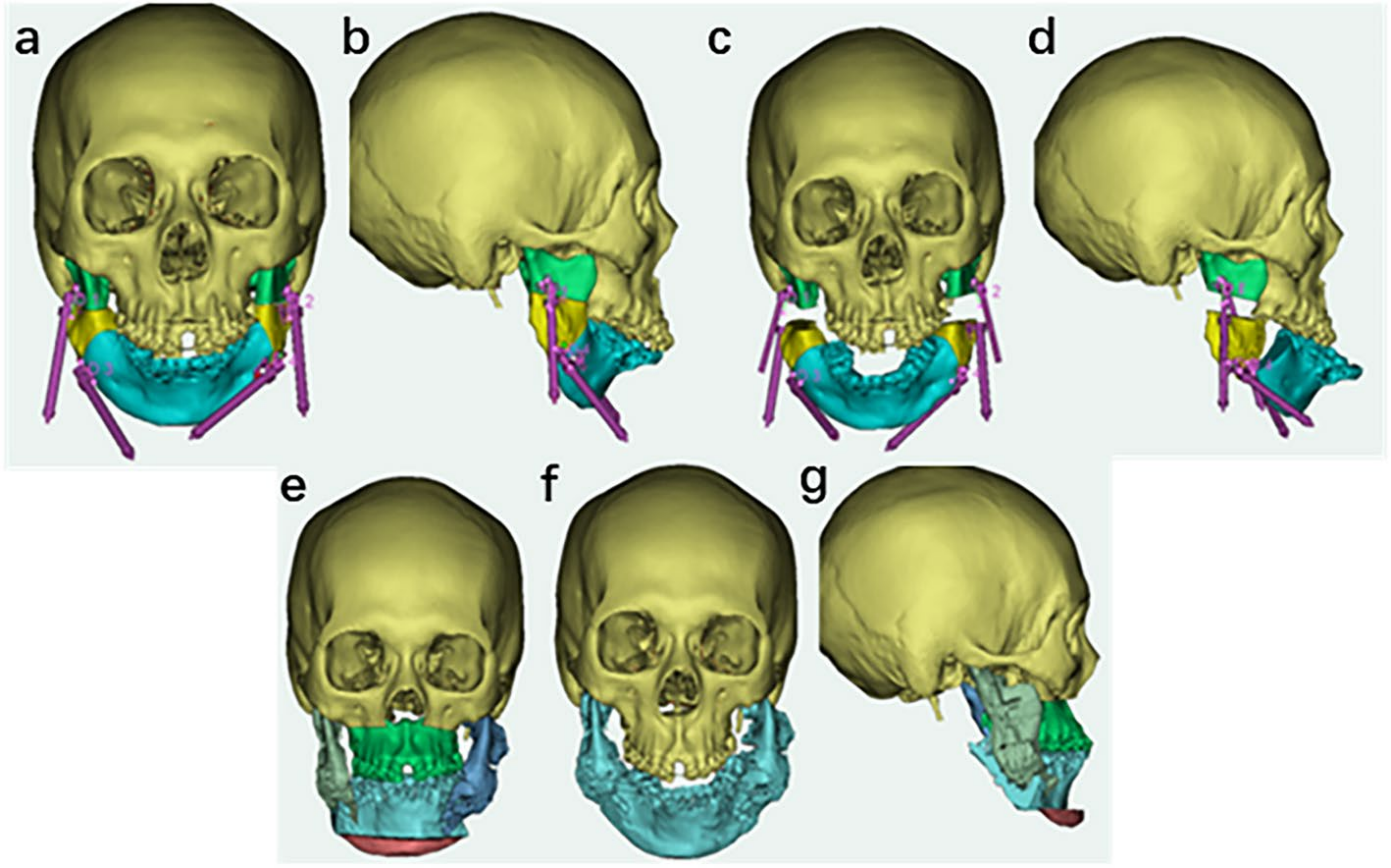
The digital virtual design for the patient’s surgery. **a**-**d** The digital virtual design for distraction osteogenesis (DO). **e**–**g** The digital virtual design for the orthognathic surgery and genioplasty

After completion of the preoperative orthodontic treatment, distraction osteogenesis (DO) was performed using a virtual surgical plan and surgical guides via a submandibular incision in July 2017. Four distractors were placed bilaterally on the mandible to lengthen the body and rami (Fig. 4a-d). After a latent period of 5 days, the distractors were activated at a rate of 0.5 mm twice daily. After the distraction phase, the left and right mandibular rums were extended by 15 and 10 mm, respectively, whereas the left and right mandibular bodies were extended by 24 and 19 mm, respectively.

Six months after DO, the patient underwent orthognathic surgery in January 2018. The patient’s front teeth were repositioned; however, the maxillary occlusal plane remained low on the left side and high on the right. A Maxillary Le Fort I osteotomy was performed to rotate the maxilla counterclockwise, achieving a central incisal cusp movement of 3 mm forward and 2 mm upward. This achieved a 4 mm drop in the mesial buccal cusp of tooth 16 and a 3 mm drop in the mesial buccal tip of tooth 26. Bilateral sagittal split ramus osteotomy (BSSRO) was performed to coordinate the jaw bones and achieve better occlusion. Finally, horizontal osteotomy and genioplasty were performed simultaneously (Fig. 4e-g).

In September 2018, the patient underwent a final surgery, including mandibular contouring and buccal fat removal, to achieve a better facial profile. The patient continued orthodontic treatment until stable and optimal occlusion was achieved in October 2019.

### Treatment results and follow-up

**Fig. 5:**
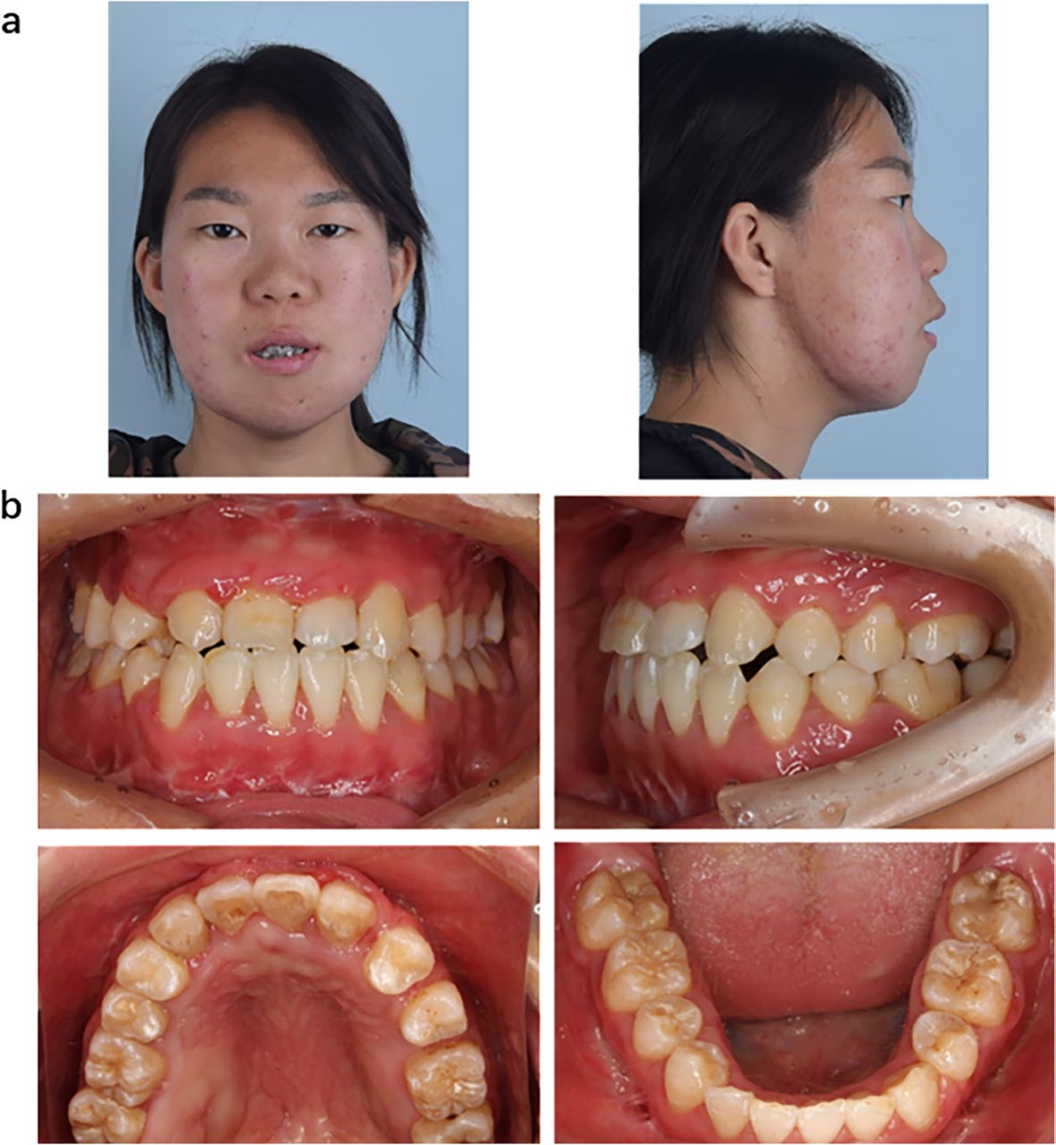
Postoperative appearance and teeth of the patient. **a** Postoperative facial appearance showing coordinated facial curves. **b** The teeth show good occlusion relation

**Fig. 6:**
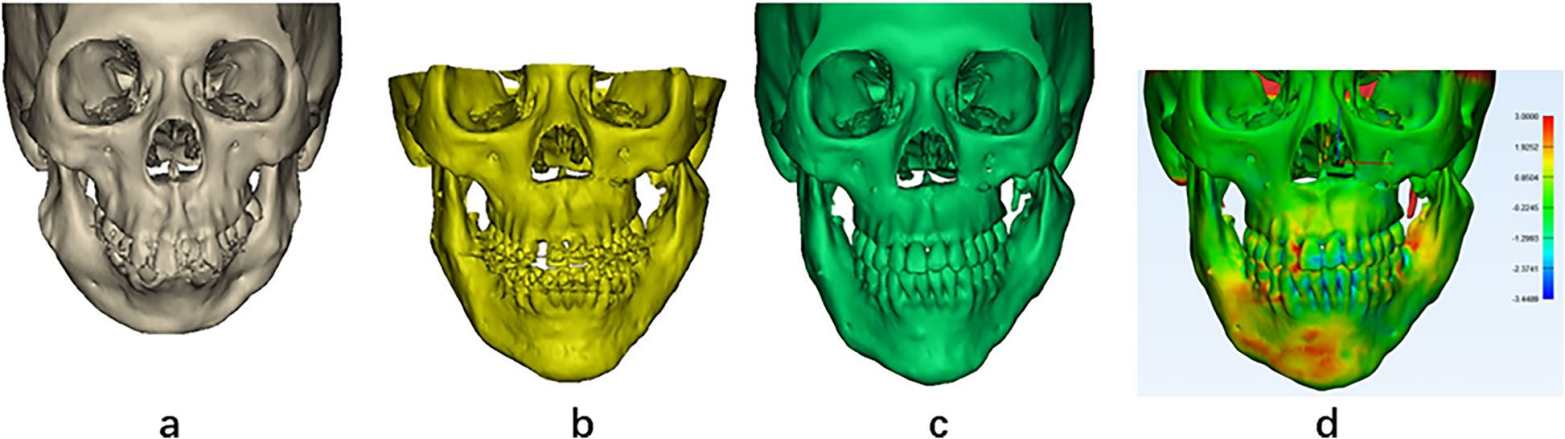
Comparison of patient's preoperative, postoperative and follow-up craniofacial three-dimensional (3D) reconstruction. **a** Preoperative. **b** Postoperative. **c** Follow-up in May 2024. **e** Fitting analysis of craniofacial three-dimensional (3D) reconstruction between postoperative and follow-up in May 2024

**Fig. 7:**
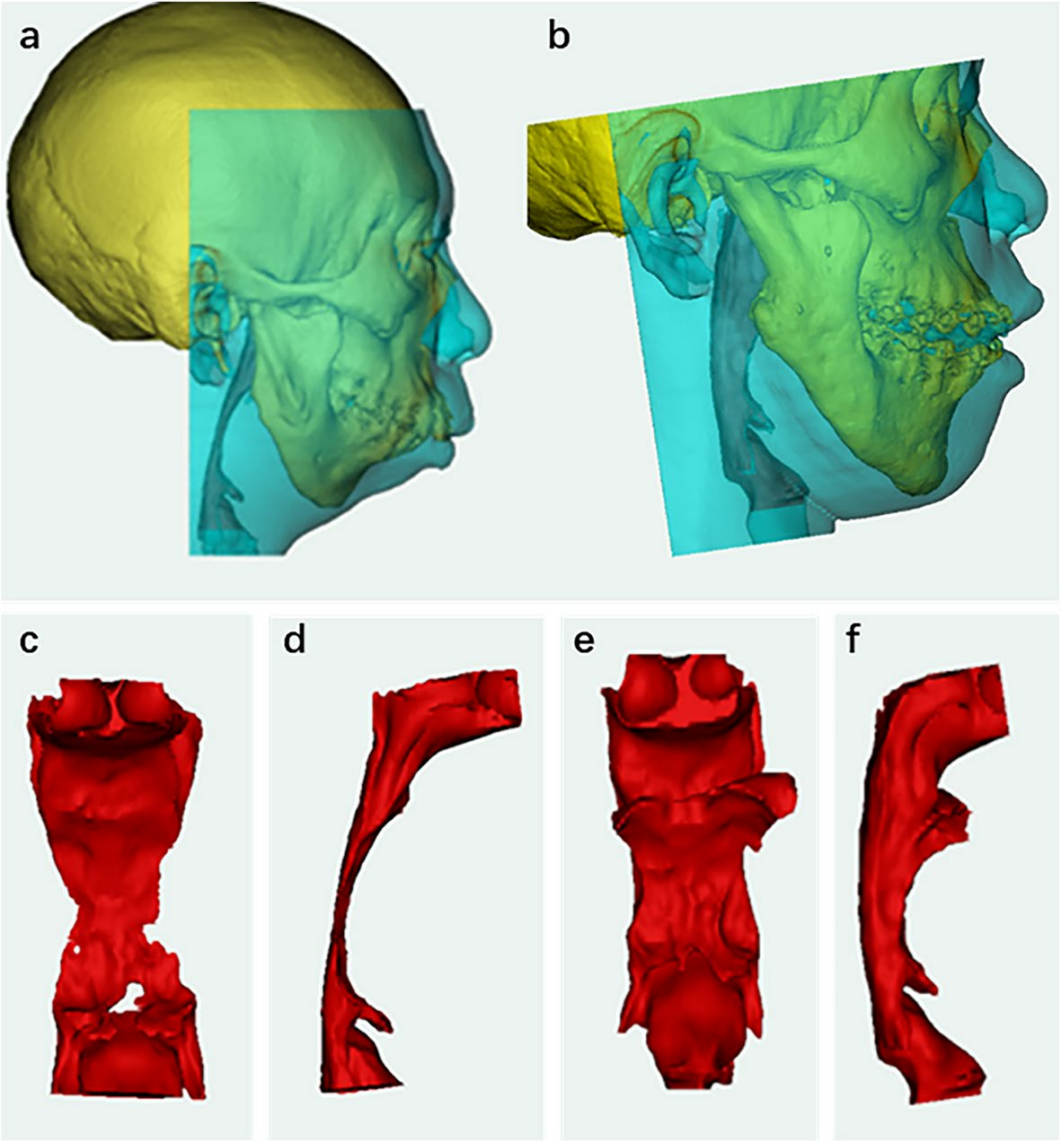
Comparison of patient's preoperative and postoperative airway. **a** Craniofacial 3D reconstruction before surgery. **b** Craniofacial 3D reconstruction after surgery. **c**-**d** Preoperative 3D reconstruction of the airway. **e**–**f** Postoperative 3D reconstruction of the airway

After treatment, the patient exhibited a symmetrical facial appearance with a well-defined facial contour (Fig. 5a). The mandible was fully extended, both horizontally and vertically, and the mismatch between the jaw and teeth was corrected (Fig. 6a, b). An approximate posterior Class I relationship was achieved, and the anterior overjet was relatively small (Fig. 5b). Marked improvement in the mandibular range of movement was observed, with a maximal opening capacity of 40 mm. The 3D reconstruction of the airway volume increased from 18,672 mm^3^ preoperatively to 32,880 mm^3^ postoperatively (Fig. 7).

The patient came to our hospital for review again in May 2024, the treatment outcome showed quite stable (Fig. 6c). Due to the incomplete symmetry and remodeling of TMJs, the mandible shifted to the right side about 1.9 mm slightly (Fig. 6d).Snoring symptoms had completely disappeared during sleep. Polysomnography revealed an apnea–hypopnea index of 0.5, and blood oxygen saturation was 97%, which demonstrated that the patient’s airway dysfunction had been significantly improved. No complications or discomfort was encountered during the follow-up period, and the patient and their family members were satisfied with the results.

## Discussion and conclusions

In 1970, Cosman et al. [12] first reported two cases of a similar deformity of the external ear and named it QMEs. In 1998, Jampol et al. [2] systematically reported the characteristic triplet features of ARCND in a family and considered it as a syndrome. Subsequently, an increasing number ARCND cases have been reported.

*GNAI3* at 1p13.3 [13, 14], phospholipase C beta 4 at 20p12.3-p12.2 [15, 16], and endothelin 1 [10, 17] at 6p24.1 are the three major deleterious gene variants of ARCND, all of which are involved in the endothelin-1-endothelin receptor type A (EDN1-EDNRA) signaling pathway [13]. An abnormal EDN1-EDNRA signaling pathway can inhibit the migration and differentiation of cranial neural crest cells, resulting in developmental defects in the first and second pharyngeal arches, ultimately causing ARCND [15, 18, 19]. Here, we report a novel *GNAI3* mutation in a Chinese family that involved the insertion of "CATTGTGAAACAGATGAA" in genes 144 and 145 in exon 2 of chromosome 110,116,384. This insertion resulted in an HCETDE segment interposing between threonine 48 and isoleucine 49, which was predicted to impair the hydrogen bonding between serine and glutamate. All these changes may potentially block the EDN1-EDNRA signaling pathway, causing ARCND. Presently, the statistics of *GNAI3* gene mutation sites included in the human gene mutation database indicate one deletion and 11 missense mutations. This new insertion mutation of *GNAI3* in ARCND has not been reported previously.

Liu et al. [1] reported that amniocentesis was performed to seek for potential genetic defects in the 30-weeks fetus with ARCND, and a heterozygous variant of c.140G > A (NM_0064) was found in exon 2 of the *GNAI3* gene by whole exome sequencing. The report showed that genetic early detection could be an effective method of early detection during the growth period. Studies about genetic early intervention during the growth period is few. After growth completion, surgical intervention is still the optimum therapeutic method for patients with ARCND so far. With the progress of gene technology, the genetic early detection and therapy of ARCND will be further developed.

The main purpose of ARCND treatment is to correct mandibular deficiency and TMJ deformity, coordinate the skull-jaw-teeth relationship, and establish good facial appearance and function. However, to date, only a limited number of sporadic ARCND cases have been reported. Ozturk et al. [20], Papagrigorakis et al. [21], Greig et al. [10], and Yang et al. [11] reported the use of a porous polyethylene prosthesis, DO, TMJ reconstruction, mandibular iliac bone transplantation, and Le Fort type I osteotomy to treat patients with ARCND. However, the application of preoperative orthodontics, DO, maxillary Le Fort I osteotomy, BSSRO, horizontal osteotomy, and genioplasty to treat adult patients with ARCND simultaneously has not yet been reported.

Aligning the teeth and adjusting the curve and width of the dental arch can facilitate the establishment of stable occlusion and improve compensatory tooth dislocation before surgery. In previous reports, we found that the height of the mandibular rami significantly increased after vertical extension. However, the length of the mandibular body remained insufficient, leading to inadequacy in the inferior 1/3 of the face and unsatisfactory condyle-to-fossa articulation. Therefore, in this case, we innovatively performed mandibular body distraction and BSSRO synergistically to coordinate the jawbone relationship and improved the functional balancing between mandibular sides and mandibular condyles. This surgical method has not been reported previously.

The correction of QMEs is another important step in the treatment of ARCND. Many surgical techniques, including double Z-plasty [12], the use of local rotation-advancement flaps [20], V–Y technique [22], use of local chondrocutaneous flaps or chondrocutaneous composite tissue transplantation [23], double-opposing Z-plasty [24], and use of cartilage grafts or expanded flaps [25], have been reported recently. These techniques have made QME treatment evidence-based and effective. Given the relatively normal deformity of the patient’s auricle in the present case, the patient declined any plastic treatment.

The application of digital technology has made the diagnosis and treatment of ARCND more controllable and predictable [9, 26]. Through 3D reconstruction of the CT data, we were able to clearly determine and analyze the cause of the deformity in this case. For example, we identified that the anterior displacement of the parotid glands and mandibular bony ridge were the primary factors contributing to the patient’s full cheeks. In addition, we observed that vertical and sagittal mandibular dysplasia contributed to airway narrowing and chin retraction. With the help of preoperative virtual design technology, we were able to execute osteotomy precisely with surgical guides and accurately design the number and position of the distraction hardware [27]. Possible issues during the distraction period can also be simulated and predicted in advance [28]. The digital design of orthognathic surgery plays a crucial role in establishing a harmonious relationship between the skull, jaw, and teeth [5, 29]. Using a 3D printed surgical guide, the virtual surgical plan was accurately replicated in the operating room, ensuring its precise implementation.

Therefore, the pathogenesis and treatment of rare hereditary maxillofacial diseases must be considered. In conclusion, we reported a novel *GNAI3* mutation case in a Chinese family and the successful treatment of dental and maxillofacial deformities with the assistance of digital technology. This study provides a reference for a better understanding of the genetic pathogenesis and practical and effective treatment of ARCND for clinicians.

## Data Availability

The complete data and materials described in the case report are freely available from the corresponding author on reasonable request.

## Abbreviations

ARCND: Auriculocondylar syndrome
QMEs: Question mark ears
*GNAI3*: Guanine nucleotide-binding protein alpha-inhibiting activity polypeptide 3
CT: Computed tomography
TMJs: Temporomandibular joints
OSAS: Obstructive sleep apnea syndrome;
3D: Three-dimensional
DO: Distraction osteogenesis
BSSRO: Bilateral sagittal split ramus osteotomy
EDN1-EDNRA: Endothelin-1-endothelin receptor type A
